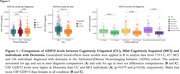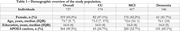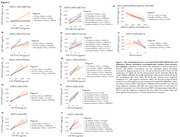# GDF15 protein as a complementary pathology biomarker of Alzheimer's Disease

**DOI:** 10.1002/alz70861_108994

**Published:** 2025-12-23

**Authors:** Rodrigo Sebben Paes, Gabriela Mantovani Baldasso, Christian Limberger, Eduardo R. Zimmer

**Affiliations:** ^1^ Universidade Federal do Rio Grande do Sul, Porto Alegre, Rio Grande do Sul Brazil; ^2^ Universidade Federal do Rio Grande do Sul, Porto Alegre, RS Brazil; ^3^ McGill University, Montreal, QC Canada; ^4^ Brain Institute of Rio Grande do Sul ‐ Pontifícia Universidade Católica do Rio Grande do Sul, Porto Alegre, Rio Grande do Sul Brazil

## Abstract

**Background:**

Growth differentiation factor 15 (GDF15), part of the transforming growth factor‐β (TGFβ) superfamily, is upregulated in response to cellular stress and various disease conditions. Elevated blood levels of GDF15 have been strongly associated with dementia, suggesting it could serve as a potential biomarker for cognitive decline. This finding is especially relevant to Alzheimer's disease (AD), where early biomarkers are crucial for improving diagnostic accuracy and enabling timely intervention. To assess its potential as a AD biomarker, this study investigate the association between CSF GDF15 levels, brain metabolism, and others AD biomarkers.

**Method:**

Data were obtained from 737 individuals from the ADNI cohort at baseline, including 174 cognitively unimpaired (CU) individuals, 417 with mild cognitive impairment (MCI), and 146 with dementia (Table 1). Generalized linear mixed models were applied to examine the association between CSF GDF15 protein levels and AD biomarkers, adjusting for age and sex in inter diagnosis groups analysis, and only for age in inter sex analysis.

**Result:**

CSF GDF15 levels is increased in individuals with dementia compared to CU and MCI individuals (Figure 1a). Across all diagnosis groups, CSF GDF15 levels were positively associated with CSF pTau181, STREM2, GFAP, and NFL (Figure 2A‐E). In MCI and Dementia group, GDF15 also showed a positive correlation with CSF ABETA42. Notably, males had higher CSF GDF15 levels than females in all groups (Figure 1B‐C), although GDF15 was strongly associated with AD biomarkers regardless of sex (Figure 2F‐J). No significant associations were found between CSF GDF15 levels and hippocampal volume and MOCA test (Figure 2K‐L).

**Conclusion:**

Our findings demonstrate that CSF GDF15 levels are elevated in MCI and Dementia compared to CU individuals and correlate strongly with key AD biomarkers, particularly pTau181, STREM2, GFAP, and NFL. The observed sex differences, with consistently higher GDF15 levels in males, suggest a potential sex‐specific trajectory in AD pathology. The lack of association with hippocampal volume and MoCA indicates that GDF15 may reflect early molecular changes rather than cognitive decline. These results support the potential of GDF15 as a complementary molecular biomarker in AD research.